# Mechanisms of Music Impact: Autonomic Tone and the Physical Activity Roadmap to Advancing Understanding and Evidence-Based Policy

**DOI:** 10.3389/fpsyg.2021.727231

**Published:** 2021-08-27

**Authors:** J. Matt McCrary, Eckart Altenmüller

**Affiliations:** ^1^Institute of Music Physiology and Musicians’ Medicine, Hannover University of Music, Drama and Media, Hannover, Germany; ^2^Prince of Wales Clinical School, University of New South Wales, Sydney, NSW, Australia

**Keywords:** non-communicable diseases, FITT, music listening, music-making, exercise physiology

## Abstract

Research demonstrates that both music-making and music listening have an ability to modulate autonomic nervous system activity. The majority of studies have highlighted acute autonomic changes occurring during or immediately following a single session of music engagement. Several studies also suggest that repeated music-making and listening may have longer-term effects on autonomic tone—the prevailing balance of sympathetic vs. parasympathetic activity. Autonomic *im*balance is associated with a range of neurodegenerative and neurodevelopmental disorders, mental health conditions and non-communicable diseases. Established behavioral interventions capable of restoring healthy autonomic tone (e.g., *physical activity; smoking cessation*) have demonstrated remarkable efficacy in broadly promoting health and preventing disease and up to 7.2 million annual deaths. Accordingly, this article proposes that music’s suggested ability to modulate autonomic tone may be a key central mechanism underpinning the broad health benefits of music-making and listening reported in several recent reviews. Further, this article highlights how physical activity research provides a relevant roadmap to efficiently advancing understanding of music’s effects on both autonomic tone and health more broadly, as well as translating this understanding into evidence-based policy and prescriptions. In particular, adapting FITT—*Frequency, Intensity, Timing, Type*—criteria to evaluate and prescribe music-making and listening in observational and intervention studies has excellent prospective utility.

## Introduction

The broad health benefits of music listening and music-making are increasingly well acknowledged (Fancourt and Finn, 2020; McCrary et al., 2021), with a recent review noting that music has been associated with positive health effects across 13 domains (*most prominently auditory, cognitive, immune, self-reported health/wellbeing, and social functioning domains*) (McCrary et al., 2021). Accordingly, the most salient questions are quickly becoming less *if* and more *how* music can be used to promote health, both in preventive and clinical/rehabilitative applications. More specifically, *what kind* of music engagement is most effective in addressing which health conditions? Additionally, *how often* and for *how long* should music engagement occur to best promote health and/or recovery in each specific circumstance?

In addressing these questions, present evidence remains limited. For instance, the recent review cited above found that as little as 30 min of music-making was associated with positive health effects in healthy populations, but could not support any further conclusions regarding the optimum music-making type, duration, frequency and health applications (McCrary et al., 2021). Such a detailed understanding is needed to inform evidence-based policy, prescriptions and care (Bickerdike et al., 2017; Clift et al., 2021), but is presently limited by common features of a young and developing evidence base: a substantial heterogeneity of interventions and outcome measures (Fancourt and Finn, 2020; McCrary et al., 2021) elucidating a glut of potential mechanisms (Fancourt et al., 2021) and leading to disparate and often contradictory outcomes (Clift, 2020; Fancourt and Finn, 2020; McCrary et al., 2021).

As a result, a strategic approach to future research is increasingly advocated to efficiently advance understanding and evidence-based policy and practice (Cheever et al., 2018; Fancourt et al., 2021). To this end, particular emphasis is being placed on theory-driven studies leveraging known mechanisms of music’s impact to guide study hypotheses, design and outcome measures toward higher probability results (Craig et al., 2008; Cheever et al., 2018; Fancourt et al., 2021). Investigations based on mechanisms linked to multiple health effects are thus likely to be particularly high-impact. With this in mind, this article focuses on autonomic nervous system mechanisms potentially underpinning many of music’s broad health effects.

Both music-making and music listening have demonstrated an ability to modulate autonomic nervous system activity (Ellis and Thayer, 2010; McCrary et al., 2021). The bulk of research has investigated acute autonomic changes during or immediately following a single session of music-making or listening, with these short-term effects linked most strongly to acute reductions in stress and anxiety, particularly in pre-operative settings (Allen and Blascovich, 1994; Bradt et al., 2013; Fu et al., 2019). Substantially less research has focused on music’s sustained impact on autonomic tone—the prevailing balance of activity in the sympathetic vs. parasympathetic branches of the autonomic nervous system (Ellis and Thayer, 2010). However, a handful of studies do suggest that repeated music-making and listening may also have a long-term effect on autonomic tone (Takahashi and Matsushita, 2006; Le Roux et al., 2007; Okada et al., 2009; Chuang et al., 2011; Helsing et al., 2016; Kunikullaya et al., 2016; Ribeiro et al., 2018; Mojtabavi et al., 2020). The potential of music to modulate autonomic tone is particularly significant given profound links between autonomic tone and a wide range of physical, mental and social health conditions (Rosengren et al., 2004; Thayer et al., 2010; Beaglehole et al., 2011; Cohen et al., 2015; Emdin et al., 2016). Further, an ability to modulate autonomic tone is a key mechanism underpinning the impact of established behavioral health interventions (e.g., *physical activity; smoking cessation*) shown to broadly promote health and prevent disease and 1.6–7.2 million annual deaths (Stein et al., 1996; Iwane et al., 2000; Curtis and O’Keefe, 2002; Fu and Levine, 2013; Harte and Meston, 2014; Forouzanfar et al., 2016).

Accordingly, we propose that music’s suggested ability to modulate autonomic tone may be a key central mechanism influencing the broad range of music’s noted physical, mental and social health benefits (Fancourt and Finn, 2020; McCrary et al., 2021). The following sections explore how investigations of music’s impact on autonomic tone can efficiently build understanding and broadly facilitate evidence-based prescriptions and policies. Additionally, later sections illustrate how physical activity research provides a relevant and adaptable roadmap to advancing and translating knowledge regarding music’s effects on autonomic tone and health more broadly.

### Autonomic Tone: A Central Mechanism of Music’s Broad Health Impact?

Briefly (*for a comprehensive summary of the physiology and function of the autonomic nervous system please see* (McCorry, 2007; Low, 2011)*; key terms are highlighted in Table 1*), the autonomic nervous system (ANS) is a division of the peripheral nervous system involved in mostly involuntary control of the major peripheral organs and organ systems (e.g., *cardiovascular, digestive, endocrine, integumentary, reproductive, respiratory, urinary, visual*). The ANS can be further divided into two main branches: a sympathetic branch broadly associated with energy mobilization (i.e., *“fight or flight” responses*); and a parasympathetic branch broadly associated with restorative and vegetative functions (i.e., *“rest and digest” responses*). Sympathetic excitation is linked to increased heart rate, blood pressure, low frequency heart rate variability and catecholamine/stress hormone release (e.g., *adrenaline; noradrenaline; cortisol*). Increased parasympathetic activation is linked to decreased heart rate and blood pressure and increased high frequency heart rate variability. In healthy individuals, the ANS dynamically responds to environmental demands before returning to a point of relative balance, stability and minimal energy use. This balance point is known as the “autonomic tone”. If one ANS branch dominates over the other (i.e., *“autonomic imbalance”*), the ability to respond to environmental situations is compromised and system energy demands are often excessive and unsustainable.

**TABLE 1:**
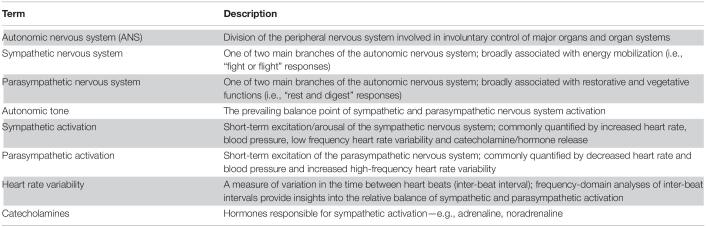
Glossary of key terms.

Autonomic imbalance is associated, as a cause and/or consequence, with a broad range of health conditions and non-communicable diseases including neurodegenerative conditions (e.g., *Alzheimer’s and Parkinson’s*), neurodevelopmental disorders (e.g., *autism*), mental health conditions (e.g., *anxiety, depression*) and cardiovascular diseases—the leading cause of global deaths (Rosengren et al., 2004; Thayer et al., 2010; Beaglehole et al., 2011; Cohen et al., 2015; Emdin et al., 2016). Autonomic imbalance is also linked to chronic inflammation (Thayer et al., 2010), a key risk factor for cancers (*#2 most prevalent cause of global deaths* (Coussens and Werb, 2002; NCD Countdown 2030 Collaborators, 2020). Fortunately, behavioral interventions such as physical activity and smoking cessation have demonstrated remarkable efficacy in resolving autonomic imbalance and restoring healthy autonomic tone (Stein et al., 1996; Iwane et al., 2000; Curtis and O’Keefe, 2002; Fu and Levine, 2013; Harte and Meston, 2014).

Music engagement has also shown an ability to positively modulate autonomic tone in several studies. Repeated engagement with music therapy including both listening and music-making components (*5 weeks to 2 years of weekly 30–45 min sessions* (Takahashi and Matsushita, 2006; Okada et al., 2009; Chuang et al., 2011; Ribeiro et al., 2018)) and recorded music listening (*3 days to 3 months of 15–30-min daily sessions* (Le Roux et al., 2007; Helsing et al., 2016; Kunikullaya et al., 2016)) has been linked to positive shifts toward increased parasympathetic tone. Increased parasympathetic tone in these studies is evidenced by changes in resting heart rate variability (Okada et al., 2009; Chuang et al., 2011; Ribeiro et al., 2018) and reduced blood pressure (Takahashi and Matsushita, 2006; Kunikullaya et al., 2016) and plasma catecholamines and stress hormones (Le Roux et al., 2007; Okada et al., 2009; Helsing et al., 2016). One study of music therapy also notably associated changes in autonomic tone with a significant reduction in heart failure events (Okada et al., 2009).

These positive results were reported in a range of populations—mothers of preterm infants (Ribeiro et al., 2018); combined cerebrovascular disease and dementia (Okada et al., 2009); breast cancer (Chuang et al., 2011); dementia alone (Takahashi and Matsushita, 2006); infectious lung disease (Le Roux et al., 2007); healthy adults (Helsing et al., 2016); and hypertensives (Kunikullaya et al., 2016). It should be noted, however, that these studies reporting positive effects of music on autonomic tone also each contain significant limitations and/or confounds—substantially underpowered (Ribeiro et al., 2018); non-randomized (Okada et al., 2009) or uncontrolled single group designs/analyses (Chuang et al., 2011; Helsing et al., 2016; Kunikullaya et al., 2016); mixed positive & null results on multiple measures of autonomic tone (Takahashi and Matsushita, 2006; Kunikullaya et al., 2016); and investigations of other interventions with or without added music listening (*physiotherapy* (Le Roux et al., 2007)*; lifestyle* (Kunikullaya et al., 2016)). Further studies have also found no effects of repeated recorded music listening (*2 days to 2 weeks of daily 30-min sessions*) on salivary and urinary stress hormone and plasma catecholamine levels (Chlan et al., 2013; Hu et al., 2015). These studies reporting null results investigated music listening in different clinical populations—mechanically ventilated (Chlan et al., 2013) and ICU patients (Hu et al., 2015)—with the study of ICU patients also notably confounded by using music and “nature sounds” as a combined intervention (Hu et al., 2015).

Well-controlled investigations of specific types of music engagement (e.g., *music-making vs. listening*) in specific populations may yield insights that help further clarify presently preliminary and limited results. However, evidence from physical activity and smoking cessation research (Stein et al., 1996; Iwane et al., 2000; Curtis and O’Keefe, 2002; Harte and Meston, 2014) indicates that such investigations would likely be still significantly confounded by a key additional variable: acute autonomic responses to music interventions. Acute autonomic responses to both music-making and listening interventions have been shown to significantly vary across individuals (Grewe et al., 2007a, b; Nakahara et al., 2009; Salimpoor et al., 2009; Chapin et al., 2010; Lynar et al., 2017). Physical activity research has effectively integrated consideration of potentially confounding acute autonomic responses into a robust body of research, prescriptions and policy related to autonomic tone and health more broadly. Accordingly, physical activity research provides a particularly relevant research and translation roadmap with prospective utility in guiding future music investigations.

### The Physical Activity Roadmap—Understanding Links Between Acute and Longer-Term Autonomic Effects

Physical activity research has developed, over 65+ years of investigations, a convincing framework for understanding the autonomic and broader health impacts of leisure-time activities and translating this understanding into evidence-based policy and care (Heath et al., 2012; Varela et al., 2018). The first major study of physical activity was published in 1953 and supported the first hypotheses that “physically active jobs” (*in this case, London postmen/bus conductors vs. government clerks/bus drivers*) may be protective against heart attacks and disease (Morris et al., 1953). Presently, a clear dose-response relationship between increased physical activity and increased quality of life and a decreased risk of early mortality and up to 25 non-communicable diseases has been established (Arem et al., 2015; Rhodes et al., 2017). Further, physical activity research has been translated into evidence-based global physical activity recommendations, prescriptions and action plans (World Health Organization [WHO], 2010; American College of Sports Medicine, 2013; World Health Organization [WHO], 2019), as well as a growing and sustaining research funding ecosystem (Fernhall et al., 2015).

A key insight informing present knowledge regarding physical activity’s extensive health effects is the impact of acute exercise-induced autonomic changes on sustained modulations in autonomic tone (Iwane et al., 2000; Curtis and O’Keefe, 2002). Similar links between acute autonomic changes and sustained modulations in autonomic tone have also been demonstrated in smoking cessation research (Stein et al., 1996; Harte and Meston, 2014). Exercise increases sympathetic activity, with the magnitude of the acute sympathetic response to physical activity shown to be reliably moderated by exercise intensity—the higher the intensity (i.e., *more vigorous the activity*), the greater the sympathetic response (Hautala et al., 2009). Broadly, the greater the cumulative weekly exercise-induced sympathetic response, the greater the impact on health and the maintenance and restoration of well-balanced autonomic tone (Arem et al., 2015; Rhodes et al., 2017).

Crucially, increased sympathetic activation during exercise also leads to a proportional increase in post-exercise parasympathetic activation, known as the “relaxation response” (Iwane et al., 2000; Curtis and O’Keefe, 2002; Fu and Levine, 2013; Rhodes et al., 2017). With regular physical activity, post-exercise parasympathetic activation becomes more robust and efficient, a change that is proposed to underpin broader improvements in autonomic tone (Fu and Levine, 2013; Pierpont et al., 2013). Music-making and listening to pleasurable and uplifting music are associated with a similar acute autonomic “relaxation response”—increased sympathetic arousal during music-making and listening to many, but not all, types of music, followed by an increase in parasympathetic activity after the music stops (Grape et al., 2002; Rickard, 2004; Bernardi et al., 2006; Grewe et al., 2007a, b; Nakahara et al., 2009; Salimpoor et al., 2009; Chapin et al., 2010; Sakamoto et al., 2013; Lynar et al., 2017). In contrast, stress and pharmaceutical-induced sympathetic excitation do not lead to substantial increases in parasympathetic activity after arousal (Watkins et al., 1998; Curtis and O’Keefe, 2002).

To efficiently capture and/or control the cumulative weekly sympathetic response, physical activity is typically assessed and “prescribed” (e.g., *in intervention studies)* according to FITT—Frequency, Intensity, Timing (i.e., *duration of each exercise bout)*, Type—criteria in observational and intervention studies (Barisic et al., 2011; Rhodes et al., 2017). FITT criteria thus consider both key practical information about the physical activity being performed (*Frequency, Timing, Type*), as well as the central mediator of the magnitude of the acute sympathetic and broader physiologic responses (*Intensity)* (Barisic et al., 2011; Rhodes et al., 2017). Inclusion of Intensity thus allows individual acute autonomic responses to physical activity to be considered and/or controlled in research and prescriptions. Both objective (e.g., *heart rate; accelerometry*) and self-report assessments have demonstrated good validity in capturing the Intensity, as well as Frequency, Timing and Type, of daily, weekly and “typical week” physical activity (Barisic et al., 2011; Rhodes et al., 2017).

FITT criteria have enabled broad research insights to be efficiently organized toward the establishment of a dose-response relationship between increased weekly aerobic physical activity (*Intensity ^∗^ Timing ^∗^ Frequency*) and improved health and reduced disease risk (Barisic et al., 2011; Arem et al., 2015; Rhodes et al., 2017). Further, this knowledge has been easily translated into policy and prescriptions (World Health Organization [WHO], 2010; American College of Sports Medicine, 2013; World Health Organization [WHO], 2019), as clearly illustrated by World Health Organization physical activity guidelines—150 min (*Timing*) of weekly (*Frequency*) moderate-vigorous (*Intensity*) aerobic (*Type*) activity (World Health Organization [WHO], 2010).

**FIGURE 1:**
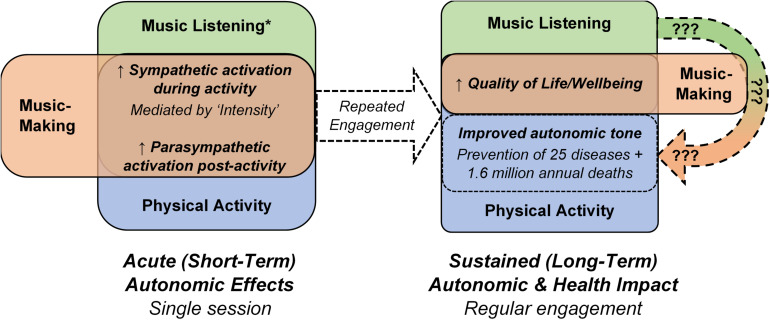
Comparison of the acute autonomic and sustained autonomic and health effects of music listening, music-making and physical activity. The unclear impact of music listening and music-making on autonomic tone and related health outcomes (Fancourt and Finn, 2020; McCrary et al., 2021) is noted by the curved arrow containing question marks. * does not apply to all types of music listening; some types of music listening elicit parasympathetic responses during the listening activity.

Similar links between acute music-induced autonomic changes and sustained modulations of autonomic tone are likely (Figure 1). However, studies of the effects of repeated music engagement on autonomic tone have only considered and prescribed FTT (*Frequency, Timing, and Type*) criteria (Takahashi and Matsushita, 2006; Le Roux et al., 2007; Okada et al., 2009; Chuang et al., 2011; Chlan et al., 2013; Hu et al., 2015; Helsing et al., 2016; Kunikullaya et al., 2016; Finn and Fancourt, 2018; Ribeiro et al., 2018; Fancourt and Finn, 2020; Mojtabavi et al., 2020; McCrary et al., 2021). Similarly, observational and intervention studies of the effects of repeated music engagement on health conditions linked to autonomic tone have not assessed and/or controlled the acute autonomic responses to music-making or listening (Fancourt and Finn, 2020; McCrary et al., 2021). These acute autonomic responses cannot be assumed to be consistent, as significant subjective variations to music engagement with identical FTT have been demonstrated—performing or listening to the same piece of music can elicit a significant sympathetic response in one individual but minimal response in another, both on average and during emotional “peaks” in the music (Grewe et al., 2007a, b; Nakahara et al., 2009; Salimpoor et al., 2009; Chapin et al., 2010; Lynar et al., 2017). Similar to physical activity, the magnitude of this acute sympathetic response during music engagement has been shown to be mediated by Intensity—music listening by emotional intensity (Rickard, 2004; Grewe et al., 2007a, b; Nakahara et al., 2009; Salimpoor et al., 2009; Chapin et al., 2010; Lynar et al., 2017), and music-making by a combination of emotional, cognitive and physical intensities (Nakahara et al., 2009; Hahnengress and Böning, 2010; McCrary et al., 2016, 2021; Yuksel et al., 2016). Further, and in contrast to physical activity, music listening has also been shown to induce a parasympathetic response during engagement, particularly during listening to relaxing music (White, 1999; Conrad et al., 2007; Kume et al., 2017); the mediators of this parasympathetic response, particularly the magnitude, are presently unclear. If proposed links between the acute and sustained autonomic effects of music do indeed exist, the present prevalence of conflicting results of studies of music’s impact on health and autonomic tone are then unsurprising (Takahashi and Matsushita, 2006; Le Roux et al., 2007; Okada et al., 2009; Chuang et al., 2011; Chlan et al., 2013; Hu et al., 2015; Helsing et al., 2016; Kunikullaya et al., 2016; Finn and Fancourt, 2018; Fancourt and Finn, 2020; Ribeiro et al., 2018; Mojtabavi et al., 2020; McCrary et al., 2021)—a large proportion of studies are likely to be confounded by an ambiguity of acute autonomic responses to the FTT of music-making and listening being analyzed.

### The Physical Activity Roadmap—Adapting FITT to Evaluate and Prescribe Music Engagement

The FITT approach carries significant promise as a means of efficiently yet comprehensively evaluating and prescribing music-making and listening to better understand their sustained effects on autonomic tone and related health conditions. Frequency, Timing and Type data would continue to provide valuable interrogative information regarding the music engagement being prescribed or analyzed. The addition of Intensity would enable individual acute autonomic responses to be considered and/or controlled in future music studies, providing more detailed insights into the mechanisms of music’s effects and a framework for standardizing a likely confounder. Given similarities in their acute autonomic responses, music-making and listening can be broadly considered under the same FITT framework. However, the practicalities of evaluating and prescribing Intensity require distinct approaches, particularly given differential Intensity mediators of the acute autonomic responses to music-making and listening (*music-making—cognitive, emotional and physical mediators; music listening—emotional mediators*) (Rickard, 2004; Grewe et al., 2007a, b; Nakahara et al., 2009; Salimpoor et al., 2009; Chapin et al., 2010; Hahnengress and Böning, 2010; McCrary et al., 2016, 2021; Yuksel et al., 2016; Lynar et al., 2017).

The absence of prior investigations evaluating or prescribing acute autonomic responses to repeated music engagement leaves a wholly blank slate for the development of new strategies. However, physical activity research and prior studies of the acute autonomic responses to music provide some guidance. The Intensity, as well as the Frequency and Timing, of music-making and listening could be evaluated using established quantitative methods with demonstrated sensitivity to the acute autonomic responses to both music-making and listening (e.g., *heart rate; heart rate variability; skin conductance; hormone, and catecholamine levels*) (Rickard, 2004; Grewe et al., 2007a, b; Nakahara et al., 2009; Salimpoor et al., 2009; Chapin et al., 2010; Hahnengress and Böning, 2010; McCrary et al., 2016, 2021; Yuksel et al., 2016; Lynar et al., 2017; Finn and Fancourt, 2018). A key distinction from physical activity with these assessments is that the acute autonomic responses will be reflective of emotional Intensity (*music listening*) or composite emotional/cognitive/physical Intensity (*music-making*), rather than the Intensity of physical exertion. The magnitude of acute autonomic responses to music-making and listening could be quantified as a% of the response at an experimentally determined peak Intensity, similar to established approaches in physical activity research (American College of Sports Medicine, 2013). New portable methods of easily and remotely collecting combined heart rate, heart rate variability and skin conductance data (e.g., Empatica E4 wristband (McCarthy et al., 2016)) enable collection of detailed objective autonomic data from an expanded range of music listening contexts. Wireless heart rate assessment is favored for capturing relatively larger acute autonomic responses to music-making due to limitations of heart rate variability in reflecting the autonomic response to increased physical intensity (Casadei et al., 1995; Polanczyk et al., 1998), in addition to the complicating effect of movement artifacts on skin conductance measurements (Dean and Bailes, 2015).

Indirect evaluation of acute autonomic responses using questionnaire assessments of Intensity has particular prospective utility in epidemiologic and observational studies, but requires additional preliminary work to ensure validity in a music context. Established FITT questionnaires from physical activity (Barisic et al., 2011; Rhodes et al., 2017), as well as music assessments addressing FTT criteria and emotional Intensity mediators of the acute sympathetic response (e.g., *Goldsmiths Musical Sophistication Index; Music USE Questionnaire*) (Chin and Rickard, 2012; Müllensiefen et al., 2014), provide an excellent foundation for the development of new self-report FITT instruments. Development processes must also consider different Intensity mediators of the acute autonomic responses to music-making and listening; additional cognitive and physical Intensity items will likely be required to completely capture the acute responses to music-making. Further, initial self-report assessments will likely be confined to Types of music listening associated with clear emotional Intensity mediators (e.g., *pleasurable and uplifting music* (Rickard, 2004; Grewe et al., 2007a, b; Nakahara et al., 2009; [Bibr B69][Bibr B10]; Lynar et al., 2017)), pending further study clarifying the emotional Intensity mediators of other music Types (e.g., *relaxing music*). Validation studies evaluating the links between questionnaire Intensity items and objective assessments of the acute autonomic response (e.g., *using methods described above*) would ensure that self-report Intensity items adequately and reliably reflect overall acute autonomic responses.

A relatively more complex task is adapting the FITT framework, in particular Intensity, to prescribe music-making and listening activities with standardized acute autonomic responses for research, clinical and general public use. In particular, development of FITT prescription strategies which translate beyond controlled research and clinical interventions to public health applications is likely to be a similarly long-term challenge for music as it was for physical activity (Varela et al., 2018). However, FITT prescriptions of music-making and listening for research and clinical interventions are more immediately feasible. To this end, we propose two examples of FITT music intervention prescriptions likely to elicit standardized acute autonomic responses while maintaining the integrity of the musical experience by keeping a focus on expression and creativity. Research demonstrates that listening to self-selected music eliciting the greatest feelings of pleasure is linked to the greatest acute sympathetic response (Salimpoor et al., 2009; Lynar et al., 2017). Accordingly, a “high Intensity” music listening prescription could be: 150 min (*Timing*) of weekly (*Frequency*) listening to self-selected music (*Type*) which will elicit the “greatest feelings of pleasure” (*Intensity*). Intervention fidelity could be tracked using objective measurement strategies described above (e.g., *Empatica E4 wristband*). In a music-making context, a prescription could be: twice-weekly (*Frequency*) 50-min (*Timing*) group drumming sessions (*Type*) at an average of 65–80% of peak composite emotional/cognitive/physical drumming *Intensity*. Peak drumming Intensity could be defined by heart rate responses and experimentally determined at the beginning of the intervention to inform target Intensity zones. Heart rate data could be collected during group drumming sessions but would not necessarily need to be monitored during sessions themselves—post-session analyses by research/clinical staff could be used to detail the observed Intensity of each session and inform modifications to future sessions to ensure consistent achievement of target Intensity by intervention participants. In both music listening and music-making FITT intervention sessions, a single blind approach to collecting acute autonomic response data is advisable to help maintain participant focus on the creative and expressive, rather than physiologic, musical experience.

## Discussion

The impact of music-making and listening on autonomic tone is potentially a key central mechanism underpinning many of music’s broad health effects—unhealthy autonomic tone is linked to a range of physical, emotional and social health conditions (Rosengren et al., 2004; Thayer et al., 2010; Beaglehole et al., 2011; Cohen et al., 2015; Emdin et al., 2016). Physical activity research provides a relevant roadmap to efficiently understanding the impact of repeated music engagement on autonomic tone, emphasizing the importance of considering and controlling the acute autonomic effects of music-making and listening in observational and intervention studies. Specifically, adapting the FITT approach from physical activity research may provide an effective framework for evaluating and prescribing acute autonomic effects alongside established moderators of music’s health impact (*Frequency, Timing, Type*) (Fancourt and Finn, 2020; McCrary et al., 2021). Over the longer-term, the FITT approach has also been shown to facilitate easy translation of research into evidence-based policy and clinical prescriptions (World Health Organization [WHO], 2010; American College of Sports Medicine, 2013; World Health Organization [WHO], 2019).

FITT evaluation/prescription of repeated music engagement must be paired, however, with sensitive and reliable autonomic tone study endpoints to most rigorously assess the impact of music engagement on autonomic tone. Physical activity research also provides valuable guidance in this domain. Specifically, four non-invasive clinical cardiac autonomic assessments have been shown to be both reliable and sensitive to intervention effects: resting heart rate; heart rate variability during seated deep breathing; chronotropic incompetence (i.e., *inability to reach 85% of maximum heart rate during exercise*); and post-exercise heart rate recovery (Tsuji et al., 1994; Lauer et al., 1996; Curtis and O’Keefe, 2002; Cooney et al., 2010; Zhang et al., 2016; Gulgun, 2017; Qiu et al., 2017). Each of these cardiac autonomic endpoints have also been independently linked to disease and mortality outcomes (Tsuji et al., 1994; Lauer et al., 1996; Curtis and O’Keefe, 2002; Cooney et al., 2010; Zhang et al., 2016; Gulgun, 2017; Qiu et al., 2017), and may provide superior utility in music investigations vs. catecholamine and hormone analyses of autonomic tone with prevailing reliability concerns (Everds et al., 2013; Segerstrom et al., 2014). However, it should be noted that these assessments are suggested to represent complementary domains of autonomic tone (Gulgun, 2017), encouraging a multimodal approach to assessment of music engagement effects, particularly in initial studies.

## Conclusion

Music and health research is presently at an exciting junction, tasked with translating strong indications of music’s broad health benefits into a specific understanding capable of supporting evidence-based public health and clinical solutions. Music’s prospective ability to modulate autonomic tone is an intriguing central mechanism potentially underpinning many reported health benefits. Established insights and approaches (e.g., FITT) from physical activity research provide valuable guidance on how understanding of music’s effects on autonomic tone can be efficiently advanced and translated into prescriptions and policies.

## Data Availability Statement

The original contributions presented in the study are included in the article/supplementary material, further inquiries can be directed to the corresponding author/s.

## Author Contributions

JMM drafted the manuscript. EA provided critical revisions. Both authors approved the final version.

## Conflict of Interest

The authors declare that the research was conducted in the absence of any commercial or financial relationships that could be construed as a potential conflict of interest.

## Publisher’s Note

All claims expressed in this article are solely those of the authors and do not necessarily represent those of their affiliated organizations, or those of the publisher, the editors and the reviewers. Any product that may be evaluated in this article, or claim that may be made by its manufacturer, is not guaranteed or endorsed by the publisher.
